# The New Chemical Nomenclature

**Published:** 1877-06

**Authors:** J. H. Logan

**Affiliations:** Professor of Chemistry in Atlanta Medical College, Atlanta, Georgia


### THE NEW CHEMICAL NOMENCLATURE.

BY J. H. LOGAN, A.M., M.D.,
Professor of Chemistry in Atlanta Medical College, Atlanta, Georgia.

Whatever the theory of a science may be, it must necessarily shape, more or less, its nomenclature. Hence the familiar use of the phrase, "the new nomenclature," to designate, par excellence, the new chemistry.

Yet it is a remarkable fact, though still not popularly recognized, that nomenclature in the new chemistry, has passed into COMPARATIVE insignificance. Why this has taken place will be explained in due time.

The popular opinion, as regards the notable revolution, or advancement, which chemistry has recently undergone, and the complexities, real or imaginary, with which it has invested the science, would be much more fully and truthfully expressed under the name of the *new notation*, than by that of *new nomenclature*, for that in connection with the novel and important theories of QUANTIVALENCE, and SUBSTITUTION, has been the real source of trouble and perplexity to students of the new books, and of confusion, worse confounded, to physicians and pharmaceutists of the old school.

Upon those who were even experts in the old forms of the written symbols and formulas of the science, it fell like the sudden sound of the barbarous jargons, heard for the first time around the tower of Babel.

It will be my aim in this brief paper, to shown first, that what is called, the New Nomenclature, is not younger than nearly one hundred venerable years, and that what is thought to be novel in it, is nothing more than a rehabilitation or simplification of all that was vigorous and admirable in one of the most perfect systems of nomenclature the world ever saw. Second, to show that when this nomenclature fails, or falls short, its place is supplied by a still more comprehensive and admirable system of notation, and that whenever this seems to present perplexing difficulties, they may be traced partly to the impatience and injustice of prejudice, and partly to the nature of the subjects embraced in the discoveries of an aggressive and rapidly advancing science.

The old nomenclature, and that which still presides over the fortunes of chemical science are identical, and it is the very same system that was devised and put in operation in 1787, by a committee of the French Academy of Sciences, at whose head were Guyon, and the celebrated chemist, Lavoisier.

At that time, and as it came from the hands of those able men, it was unsurpassed, except in the exact sciences, as a system of terminology. Despite some serious faults, its merits were universally acknowledged, and the valuable results, which it has achieved since for the science, are demonstrative proofs of its great practical value.

Whatever simplifies a science, elevates and advances it, and if this can be claimed for the new chemistry through its recently modified nomenclature, it will be sufficient to show the fact, in order to give it a new and favorable character in the popular regard.

But it has done more than merely simplify; it has corrected in good part, the evil tendency of the false dualistic theory of Lavoisier, and his co-laborers, at the same time adapting itself to the advanced theories of the science, and in some part, even to the novel and extraordinary developments in the dominion of organic chemistry.

The French chemists of 1787 crowned oxygen as the great leading, central figure of chemical art and science. Theirs was the chemistry of oxygen, and this ruling idea gave both form and character to their nomenclature. Oxyge nhad chemical affinity for every other element, save one; oxygen entered, as prominent factor into the generation of nearly all the most important acids, and displayed, in the act of its numerous combinations, an energy and brilliancy which elicited the involuntary homage of both scientists and people. Oxygen was KING.

But like his great contemporary, King Cotton, of the agricultural section of the United States, ninety years of experience and development were too much for the stability of his throne; the logic of circumstances, most of all, of rapidly unfolding scientific phenomena, overwhelmed him, and no charming priestess cheered by her presence and the pomp of ceremonial rites his passage to a lower seat.

In the new chemistry hydrogen is KING. And, like true greatness, but unlike oxygen, it passed up to its new honors without a formal coronation, or even a flourish of trumpets. Its claims had become numerous and urgent.

Hydrogen is the lightest of all known substances; of all the elements, hydrogen possesses the most intense CHEMISM; hydrogen is the great standard of density, of atomic and molecular weight, of molecular volume, and chemical quantivalence; and, last, but not least, HYDROGEN, and not oxygen, is the universal acid generator.

Given the scientific facts of the new chemistry, and deduct ninety years from the era, and what is now known as hydrogen, might have been denominated oxygen, and the latter element, with propriety, could have been named hydrogen, for an invariable product of all ordinary combustion is water, and all ordinary combustion is but the combination of oxygen with the burning substance.

A change so complete as this, would, in government, be revolution; in chemistry, it required such a modification of the entire system of nomenclature as would adapt it to the new condition of things.

The situation and the problem before modern chemists, were simply these: first, The youth had outgrown his clothes—his healthy, vigorous proportions had expanded far beyond the protective capabilities of the garb given him by his illustrious father; second, What is the remedy? for it will not do to allow the boy to go about thus neglected and half naked.

And, if it is decided to give him another decent suit, how shall it be done? Will it be best to patch up the old, outgrown clothes, or to cast them away entirely, and put upon him a wholly new outfit? There were insuperable difficulties to the last proposition.

The old dualistic system of Lavoisier, imperfect and false, as it had been from the first, in theory, had become so encrusted, as it were, upon the forms and expressions of the science, and, withal, possessed so many admirable excellencies that it was impossible to throw it wholly aside for another, even though it were seriously attempted.

To patch the old system, and make the best of it, was, therefore, the only practical answer to the problem. And this is precisely what was done.

One of the first steps taken towards reforming the old nomenclature, was, to yield some of the arrogance of its pretensions to the superior capabilities of notation. Long after the death of its great author, it continued to aspire to a sort of universal dominion over the science of chemico-physics. Nothing was too complex for its methods, or too subtle for its flexibility.

Its self-conceit was akin to that of the famous painter, who boasted that he was able, with his facile brush, to give expression to any conception ever embodied in words, till reminded by another that Job has clothed the neck of the war-horse with STRENGTH and THUNDER.

It assayed to express, in convenient, euphonious language, the most complex molecules, and was ambitious of popular favor, even while elaborating such useless barbarisms as follow: $H_2 Na P O_4$, Dihydric-sodium phosphate, $Na_2 B4 O_7$, Disodic-tetraborate, $H Na_2 P O_4$, Hydric-di-sodium phosphate, and $H_4 Ca (P O_4)_2$, Tetrahydro-calcic-di-phosphate.

It was this pedantic jargon, with other facts, now quite apparent, which first gave evidence of inherent weakness in the system, and when, at length, the later investigations in the domain of organic chemistry, opened wide the flood-gates to endless compounds of teeming atoms, the pressure became too great for any mere pretension, and concession and reform were the order of the day.

The next commendable improvement of the system was its reduction to a form more simple, convenient and consistent, as a whole. This is seen in the fact that a simple principle and rule are made sufficient for its constitution and authority, while a few modifications of the same rule extend and complete its application.

Lavoisier, himself, laid down the principle, to-wit: THAT THE NAMES OF ALL COMPOUND MOLECULES SHALL BE DERIVED FROM THOSE OF THEIR CONSTITUENT ATOMS. The rule may be stated thus: PLACE THE NAME OF THE POSITIVE CONSTITUENT FIRST, AND AFTER IT THAT OF THE NEGATIVE WITH THE ENDING IDE. This is applied primarily to binary compounds, but with a few simple changes, as regards the terminal ide, this rule is made applicable to every compound, whether acid, base, or salt—ternary, or quarternary molecules.

A simple rule, therefore, and a homœopathic pill box collection of prefixes and suffixes, constitute the entire outfit of the new nomenclature. The prefixes are per, hypo, di, bi, tri, tetra, penta; the suffixes, ic, ous, ate, ite, ine, yl, ide. By the old system, a name was written in one order and read in another. FeO was so written, and read PROTOXIDE OF IRON; now, to be more simple and consistent, it is written and read in the same order, FEROUS OXIDE, and without the old prefix pro $Fe_2$ $O_3$, sesque oxide of iron, or the per oxide of iron, of the old, is simply FERRIC OXIDE of the new. The prefixes, sesque, proto and deuto, are obsolete.

For the sake of euphony, and also to secure the advantage of a universal language, adjectives are used formed from the Latin names of the positive elements of compounds. $Ag_2$ O, is argentic oxide; Pb O is plumbic oxide, and Sn $O_2$ is stanic oxide.

If the same element forms with oxygen two compounds, the suffix *ic*, is used for the higher oxide, while the terminal *ous*, designates the lower.

Thus Fe O is Ferrous Oxide; $F_2$ $O_3$ is Ferric Oxide; $N_2O$ is Nitrous Oxide; and $N_2$ $O_2$ is Nitric Oxide.

When, in any case there are more oxides than two of the same positive element; or if there be any inconvenience in the use of the adjective ending *ous*, they are distingushed by the Greek numeral prefixes; di. tri. and tetra; $N_2$ $O_3$ is Dinitric Trioxide; N $O_2$ is Nitric Dioxide; $N_2$ $O_5$ is Dinitric Pentoxide.

It is no small advantage, moreover, gained by these numeral prefixes, that they extend the principle of Lavoisier, by giving in addition to the names of ingredients, clearly and easily, and within a large range, the exact proportions of each in any compound.

The old prefixes sub, proto, and deuto were a constant source of misapprehension to the student, for it was difficult for him to see why, and to remember, they did not refer to the number of atoms supplied by the negative ingredient, but designated only a certain order of combination, that is, first and second unions of the same elements. This deficiency is fairly removed by the new arrangement. Now, $Cu_2O$ formerly read sub-oxide of copper, is read cuprous oxide, and Cu O once read protoxide of copper is spoken cupric oxide, by a slight modification of the general law already given.

But the improvement is not confined to binaries; it extends to ternaries as well—to salts and acids. Where an acid, for example, combines with an *ous* base or oxide, it is not named a proto-sulphate, or proto-nitrate as the case may be, but simple an *ous* salt. Fe O united with sulphuric acid, is ferrous sulphate, and not proto-sulphate of iron, as formerly, and $Fe_2 O_3$ with the same acid, is ferric-sulphate instead of per or sesqui-sulplate. Nothing can be plainer or simpler than this.

We remarked that for certain reasons, the Latin names of elements were used in the construction of those of compounds; yet $Ag N O_3$ is not read argentous-nitrate, but silver-nitrate, for the simple reason, that silver, as an adjective, is more euphoneous and convenient than argentous. There are other examples of the same kind. Both C O and $C O_2$ have the adjective *carbonic* before the negative ingredient, since carbonous is not so smooth or convenient a word; they are carbonic dioxide and carbonic acids.

A characteristic of the terminal *ide* is worthy of special notice; it invariably indicates a compound of only two elements.

The method of naming higher compounds of three or more elements, such as the acids, salts and bases, is precisely analogous to that of the binaries, through the operation of the simple rule already given. By far the greater number of the mineral acids are compounds of hydrogen and oxygen united with some third element. This third ingredient gives the compound its distinctive character, and consequently its name; precisely, therefore, as in the *ide* molecules, a greater or less quantity of oxygen in the compound is indicated by the suffixes *ic* and *ous*.

$H N O_3$ is Nitric acid: $H N O_2$ is Nitrous acid; $H_2 S O_4$ is Sulphuric acid; $H_2 S O_3$ is Sulphurous acid.

Now there are several processes by which the hydrogen in any acid may be replaced by a positive metal forming these, by a large, and important class of compounds known as salts. Here again, the system is true to simplicity and analogy; the generic name of the salt is formed from that of the acid, by changing the terminals *ic* or *ous* into *ate* or *ite* according to the acid used. Phosphoric acid form phosphates; phosphorous, phosphites; carbonic acid, carbonates; sulphuric acid, sulphates and sulphurous acid, sulphites.

As before, the several salts of the same acid are distinguished by adjectives formed from the name of the positive ingredient.

If the acid be $H N O_3$ or nitric acid, and the metal, sodium, then the salt will be sodic nitrate; if silver, argentic nitrate, if coper, cupric nitrate, etc. But if the acid be $H N O_2$ nitrous acid, the same metals, in succession, may form sodic nitrate; argentic nitrite, and cupric nitrite. And again, as in the oxides, where there is more than one equivalent of oxygen in the base, the suffixes *ic* and *ous* are in easy requisition, and we have $Fe S O_4$ ferrous sulphate and $Fe_2 S_3O_4$ ferric sulphate, $Hg_2S O_4$ mercurous sulplate, and $Hg S O_4$ mercuric sulphate.

When the name of the positive element of the base does not afford an agreeable objection in *ous*, then an *ic* adjective is used in any equivalency of the negative, or the original name unchanged. Hence, $K_2O$ is read potassium monoxide.

$K_2O_2$ potassi*um* dioxide, and $K_2O_4$ potassi*um* tetroxide or potass*ic* in each case, and not potasseous. The same remark may be of sodium; $Na_2$ O is sodi*um* monoxide, and $Na_2O_2$ sodi*um* dioxide or sod*ic* in each case.

But an acid is also, a salt, and may be named as such. $H N O_3$ is, therefore hydric nitr*rate*, and $H N O_2$ hydric nitrite. These are really the chemical names of those acids, while those already given above, are but their common, every day names; that is nitric and nitrous acid.

An acid may be readily converted, not only into a salt, but also into a base, and a base into an acid again, or salt. Acids, bases and salts, therefore, do not differ from each other, neither in their nature, whatever that may be, nor molecular form.

This is, doubtless, the most distinctive feature of the new chemistry, and is in close analogy with that most remarkable scientific generalization of the age, the CORRELATION OF ENERGY in the world of physics. Mechanical force is convertable into heat, heat into electricity, and this again into mechanical force and heat, and the latter, once more, into mechanical energy. Whatever, therefore, is the essential nature of one of these physical forces, it is identical with that of all the rest.

These facts, as regards, especially, the last mentioned chemical compounds, have materially modified the nomenclature. $Na_2\ SO_3$ is a salt, sodic sulphite, in which two atoms of hydrogen in the acid, have been replaced by two equivalents of sodium, and $H_2\ SO_3$, the acid from which it is derived. Sodic, or sodium sulphite, is a salt in which two other atoms of hydrogen, in two equivalents of water, have been replaced by the negative $SO_2$ forming sulphurous acid.

$H_2\ SO_4$ is hydric sulphate, or an ate salt from the same water base; $H_2\ S_2\ O_3$, hydric hyposulphite, or hyposulphurous acid.

Take $Na_2\ SO_3$ again, replace its two atoms of sodium with as many of hydrogen, and it is once more $H_2\ SO_3$, an acid, without the least change of molecular form.

$Na_2\ SO_3$ is a normal salt that is perfectly neutral as regards acid or basic properties. Now replace but one of the two hydrogen atoms of the acid $H_2\ SO_3$ with as many of the sodium, and there appears an acid salt whose symbol is H. Na $SO_3$, hydrosodium sulphite.

Take the base K O H, potassium hydrate, another salt from the water base, replace its hydrogen atom with the negative radical, N $O_2$, and there results K N $O_3$, potassic nitrate, or a base changed to a salt.

The double salts, whose names were so ill-provided for by the old nomenclature, are especially indebted to the simplicity and euphony of the new. Na Ca Sb $O_4$, is sodio-calcium antimoniate. $Na_2\ Al_2\ Cl_8$, is sodio-aluminum-chloride; and $Al_2\ K_2\ (S\ O_4)_4 x_{24}\ H_2\ O$, the well known complex molecule of common alum, is read, patassio-aluminic sulphate.

The names acid, salt and base, it is obvious, have lost all distinctive, or technical meaning in chemistry. They are only necessary now from having been fixed, or crystalized, as it were, in usage, from long popular habit and scientific recognition. They are consigned to the same category with such terms as IDEA in intellectual philosophy, CURRENT and FLOW, NEGATIVE and POSITIVE, in electricity, and INCIDENCE and REFLECTION in optics—SCIENTIFICALLY black-balled, but PRESCRIPTIVELY and TRADITIONALLY enthroned forever.

The proposition in regard to notation, at the head of this paper must be reserved for another number of the JOURNAL.

